# The effect of neuromuscular re-education and oral motor exercises on adaptation and masticatory performance in complete denture wearers- a systematic review

**DOI:** 10.3389/fdmed.2026.1949919

**Published:** 2026-09-01

**Authors:** Diya Shetty Ganesh, Shipha Hegde, R. Nirupama

**Affiliations:** Nitte (Deemed to be University), AB Shetty Memorial Institute of Dental Sciences (ABSMIDS), Department of Prosthodontics and Crown and Bridge, Mangalore, Karnataka, India

**Keywords:** biofeedback, complete denture, functional adaptation, masticatory function, neuromuscular re-education, oral motor exercises

## Abstract

**Purpose:**

This systematic review aimed to evaluate the effect of neuromuscular re-education, oral motor exercises, or biofeedback compared to standard post-insertion care on objective masticatory function and patient-reported outcomes in completely edentulous adults receiving new conventional complete dentures.

**Methods:**

A pre-piloted search protocol was developed in accordance with the Preferred Reporting Items for Systematic Reviews and Meta-analyses – Protocol guidelines (PRSIMA-P) and registered in PROSPERO (CRD420251186130). A comprehensive electronic search was conducted across various databases. The selected studies were further evaluated using the Cochrane ROB 2 tool for randomized controlled trials (RCTs) and the ROBINS-I V2 tool for non-randomized studies.

**Results:**

The initial search yielded 1,517 records from which a final set of 3 articles (2 RCTs and 1 clinical trial) were included. Quality assessment indicated that one RCT had “some concerns,” one RCT was “high risk,” and the clinical trial was at “critical risk” of bias. A sensitivity analysis suggested that excluding the higher-risk studies did not alter the overall positive direction of the findings. The findings of the included studies indicated that oral motor exercises and biofeedback significantly improved the functional adaptation as well as patient satisfaction.

**Conclusion:**

The presented evidence suggests that active neuromuscular training is a cost-effective and feasible intervention to improve the functional adaptation to conventional complete dentures. Further high-quality research is recommended to substantiate these findings and to develop a protocol advocating for oral motor exercises and biofeedback to be included as a part of post-insertion instructions for conventional complete dentures.

**Systematic Review Registration:**

PROSPERO(CRD420251186130).

## Introduction

Oral rehabilitation plays an essential role in restoring the functional health of the masticatory system and improving the individual's quality of life (1). The established standard of care in functional rehabilitation of the oral cavity has been complete dentures for many decades, with varied techniques utilized to achieve optimal function and aesthetics. While significant advancements have been developed in this field such as implant-retained prostheses (overdentures and hybrid prosthesis) (2, 3), conventional complete dentures remain a primary treatment modality. This is driven by various factors such as financial constraints, reluctance of patients in undergoing invasive therapy, anatomical limitations for implant placement, and the relative simplicity of the conventional prosthesis, further contributing to patient comfort and satisfaction (4, 5).

The clinical success of a complete denture can be ascertained by its fit, defined in terms of retention and stability. Retention refers to a denture's resistance to vertical displacement, which relies on the vertical masticatory movements of the patient. Stability pertains to its resistance to horizontal displacement, a factor determined by the functional pressures exerted by the patient's oral musculature (6). These mechanical properties are dependent on the quality and the quantity of the residual alveolar ridge. The absence of adequate retention and stability could significantly impact the masticatory function, leading to denture soreness, mucosal irritation, and further deterioration of the patient's oral health related quality of life (OHRQoL).

However, in conditions of complete edentulism, the challenge of rehabilitation goes beyond mechanical factors. It also represents a significant sensory deficit which requires rehabilitation. The loss of natural teeth is accompanied by the loss of periodontal mechanoreceptors which play an essential role in the proprioceptive mechanism of the masticatory system (7). In such conditions, rehabilitation of the oral cavity requires re-orientation of the patient's mechanoreceptors, shifting from reliance on periodontal mechanoreceptors to those of the mucosa and associated tissues (8). This physiological shift results in development of new motor skills wherein the patient has to essentially re-learn and re-orient themselves to learning the basic essential functions such as chewing and speech.

Thus, beyond mechanical factors, successful oral rehabilitation is dependent on the patient's neuromuscular adaptation to the prosthesis. A technically accurate prosthesis cannot successfully re-establish the masticatory function without functional adaptation, i.e, the patient has to develop a new set of co-ordinated motor skills to effectively achieve complex movements such as speech and mastication without displacement of the denture (9). While most patients adapt over time with passive learning, a significant subset fail to develop the necessary neuromuscular coordination. This often leads to lowered usage of the dentures, avoidance of social eating, dietary compromises, failure to achieve functional usage of the dentures, leading to personal dissatisfaction (10, 11).

To bridge this gap, interventions such as oral motor exercises (myotherapy), chewing training, and biofeedback have been proposed to accelerate this neuroplastic adaptation (12, 13). It is hypothesized that these structured training programs re-educate the orofacial musculature, leading to faster functional improvements and higher satisfaction. Currently, the evidence supporting these interventions is scattered across various small clinical trials. No high-level systematic review has synthesized this data to determine if these exercises provide a quantifiable benefit over standard post-insertion instructions. This review aims to fill that gap by evaluating the effect of these interventions on both objective (masticatory) and subjective (patient-reported) outcomes.

## Materials and methodology

### PICOS question

The population (P), intervention (I), comparator (C), outcome (O) study design (S) framework was chosen for the present review and the following question was formulated: “In completely edentulous adult patients receiving new conventional complete dentures (Population), what is the effect of neuromuscular re-education, oral motor exercises, or biofeedback (Intervention) compared to standard post-insertion care (Comparator) on objective masticatory function and patient-reported outcomes (Outcome) as observed in randomized and non-randomized clinical trials (Study Design)?”

### Eligibility criteria

The following criteria were utilised to determine inclusion of studies in the present review:

#### Inclusion criteria

- Studies evaluating completely edentulous adults (any age/gender) receiving new complete maxillary and mandibular dentures.
- Interventions involving structured neuromuscular training (e.g., oral motor exercises, reading aloud, biofeedback).
- Studies comparing the intervention to standard care or no intervention.
- Randomized Controlled Trials (RCTs) and Clinical Controlled Trials (CCTs).
- Studies published in the English language.

#### Exclusion criteria

- Studies involving implant-retained or -supported overdentures.
- Studies focusing on denture relines, repairs, or replacement of old dentures without new fabrication.
- Studies reporting on individuals with temporomandibular disorders (TMD) or significant neuromuscular pathology (e.g., stroke, Parkinson's) unless specifically targeted for denture adaptation.
- Single case reports, literature reviews, editorials, *in-vitro* designs, and animal studies.

#### Search strategy

A comprehensive literature search was designed based on a pre-piloted protocol registered in the International Prospective Register of Systematic Reviews, PROSPERO (CRD420251186130) (14). The protocol was designed as per the Preferred Reporting Items for Systematic Review and Meta-Analysis Protocols (PRISMA-P) 2015 guidelines (15). This review adhered to the PRISMA 2020 guidelines for conducting systematic reviews (16).

A unique search strategy was designed for the individual databases (PubMed/MEDLINE, Scopus, Cochrane Central Register of Controlled Trials, Web of Science) using a combination of Medical Subject Headings (MeSH) terms and common keywords in conjunction with the Boolean operators (AND, OR, NOT). Additionally, a grey literature search was performed using Google Scholar to identify relevant theses and conference proceedings. Following the search, the collected results were analyzed for inclusion as per the pre-defined eligibility criteria with the aid of Rayyan software (17). Additional articles were identified via hand-searching and manual screening of the references from the included articles (backward snowballing). The title and abstract screening were conducted by two autonomous reviewers following which the included articles underwent a full-text review for further assessment of eligibility. Any disagreements were resolved with mutual discussion.

#### Quality assessment of the included studies

The study designs of the included studies were considered and individual risk of bias assessment was executed with tools specific to the same. Randomized Controlled Trials (RCTs) were evaluated using the Cochrane Risk of Bias 2 (RoB 2) tool (18). Non-randomized studies (CCTs) were evaluated using the ROBINS-I V2 tool (19). The studies were graded as having ‘Low Risk', ’Some Concerns', ‘High Risk', or ‘Critical Risk' of bias based on the domain-specific algorithms of the assessment tools.

#### Data extraction

Specific data were obtained from each individual study and a summary of the included studies was compiled including: Author, year of publication, country, study design, population characteristics (sample size, age, sex), intervention details (type, frequency, duration), comparator details, outcome measures (primary and secondary), and key findings (Supplementary Table S1). The data was compiled using a Microsoft Excel spreadsheet.

## Results

### Study selection and characteristics

An initial set of 1,517 records were identified from the databases. After removing duplicates and applying automated filters, 531 records remained for title and abstract screening. Of these, 526 were excluded as they did not meet the PICO criteria. Five reports were sought for retrieval and assessed for full-text eligibility. Two studies were subsequently excluded due to ineligible interventions (prosthetic modifications) or ineligible populations. Ultimately, three studies met all eligibility criteria and were included in the systematic review.The characteristics of the included studies are summarized in Supplementary Table S1 and Supplementary Table S2.

### Quality assessment of the included studies

**Figure 1:**
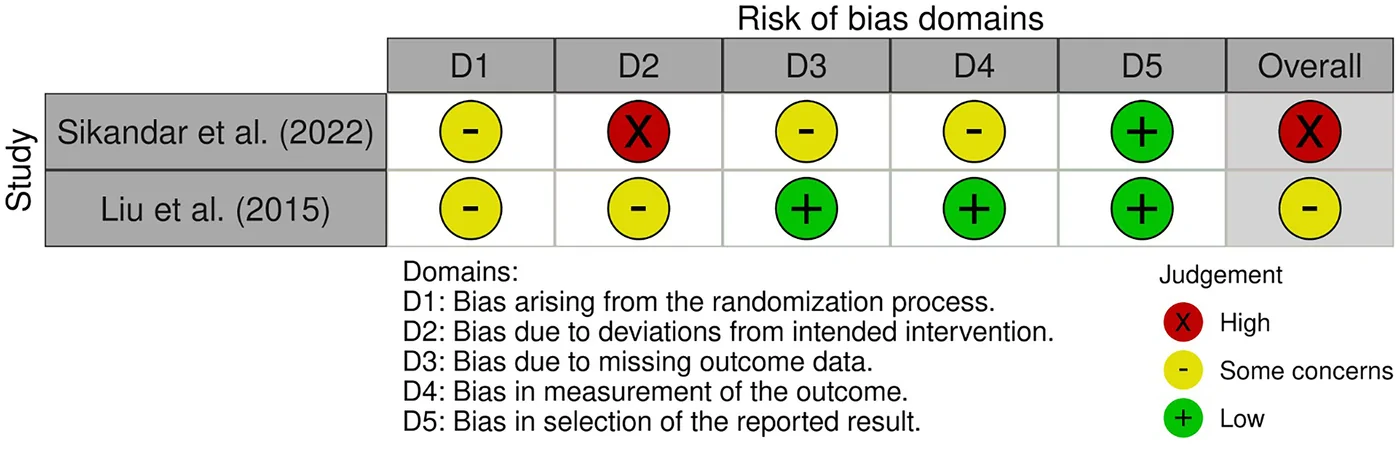
Risk of bias assessment using the cochrane ROB 2.0 tool.

**Figure 2:**
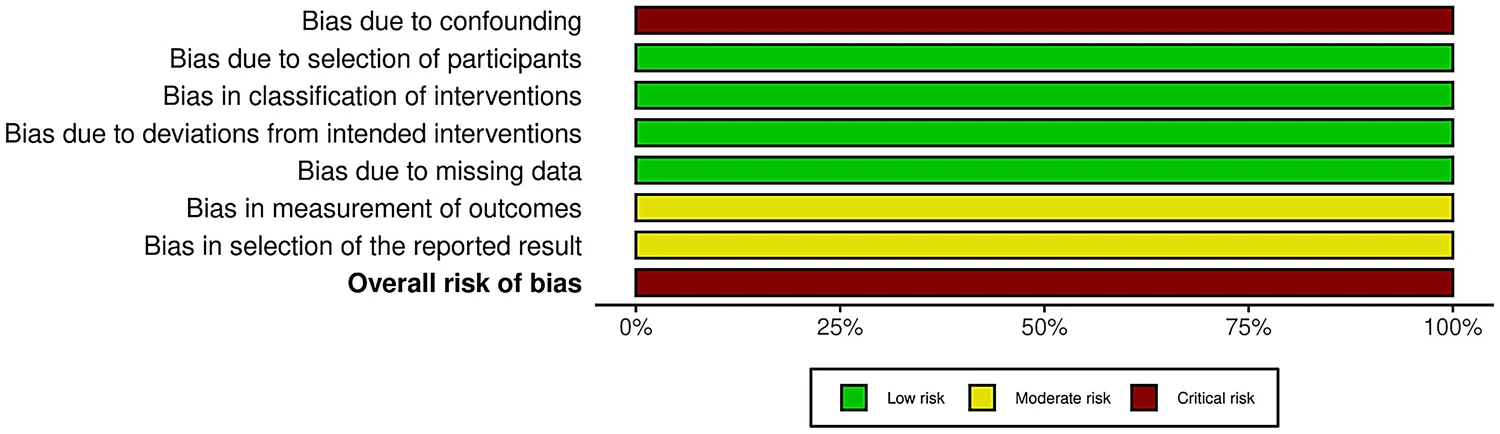
Risk of bias assessment using the cochrane ROBINS - 1 tool.

Quality assessment stratified the included studies into varying levels of bias (Figures 1, 2). Among the RCTs, Liu et al. (2015) (12) was assessed as having “Some Concerns,” primarily due to the lack of participant blinding inherent to behavioral interventions, though outcome assessment was blinded. Sikandar et al. (2022) (13) was assessed as “High Risk” due to concerns regarding randomization concealment and lack of blinding. The non-randomized study, Yemm (1983) (20), was assessed as “Critical Risk” using ROBINS-I due to critical confounding between the symptomatic intervention group and the asymptomatic control groups.

### Study outcomes and measures

The included studies were conducted in Pakistan (13), China (12), and Scotland (20), with a total pooled sample of 147 participants. The interventions were classified into two main categories: Active Oral Motor Exercises (specifically reading aloud) and Biofeedback.

### Masticatory performance and muscle activity

All three studies reported positive outcomes regarding objective function. Both RCTs utilized a “reading aloud” exercise protocol. Sikandar et al. (2022) reported that the exercise group achieved a significantly higher mean masticatory performance score (37.13% ± 1.47) compared to the control group (29.60% ± 4.29) at 4 weeks post-insertion (*p* < 0.001) (13). Similarly, Liu et al. (2015) found that the reading aloud group demonstrated superior masticatory performance (47.60% ± 3.28) compared to controls (38.99% ± 3.07) at the 4-week follow-up (*p* < 0.001) (12). Yemm (1983) reported that biofeedback training significantly reduced resting temporal muscle activity (EMG levels) in patients with persistent denture soreness (20).

### Patient-reported outcomes

Patient satisfaction was evaluated in one study. Liu et al. (2015) assessed satisfaction using a Visual Analogue Scale (VAS) (12). At 4 weeks, patients in the reading aloud group reported significantly higher satisfaction scores regarding denture stability, ability to speak, and ability to chew (*p* < 0.001) compared to the control group.

### Sensitivity analysis

A narrative sensitivity analysis was performed to evaluate the impact of the studies with high or critical risk of bias (13, 20). Excluding these studies did not alter the primary direction of the findings. The remaining study with “some concerns” provided consistent, statistically significant evidence regarding the benefits of oral motor exercises on masticatory performance and patient satisfaction (12). Consequently, the inclusion of the lower-quality studies did not skew the overall qualitative synthesis, although their magnitude of effect should be interpreted with caution. A narrative sensitivity analysis was undertaken to examine the robustness of the direction of the findings to the inclusion of studies judged to have high or critical risk of bias. The qualitative synthesis was first considered including all eligible studies and was then repeated after excluding studies assessed as having high or critical risk of bias. The direction and consistency of the findings across the remaining evidence were compared descriptively. No statistical sensitivity analysis was performed because quantitative meta-analysis was not feasible.

## Discussion

### Summary of evidence

This systematic review aimed to evaluate the efficacy of neuromuscular re-education and oral motor interventions on the functional adaptation of new complete denture wearers. The synthesis of the included studies, comprising two randomized clinical trials (12, 13) and one clinical controlled trial (20) provide consistent evidence that active neuromuscular training significantly improves masticatory function. Furthermore, evidence from one of the assessed studies indicates that the improved function further translates to increased patient satisfaction and improvement in additional oral functions such as speech (12).

### Interpretation of findings

From the findings of the present study, it may be hypothesised that the improvements noted can be attributed to accelerated tissue neuroplasticity. As previously discussed, successful adaptation to complete dentures requires the re-direction of the lost periodontal mechanoreceptor feedback to mucosal and muscular proprioception (21). This may not be achieved with standard post-insertion care which is reliant on passive habituation, a slow and variable process.

#### Oral motor exercises

The “reading aloud” protocol employed by Sikandar et al. (2022) (13) and Liu et al. (2015) (12) train the orofacial musculature to actively co-ordinate during complex movements of speech and maintain denture stability. This redirection of muscular forces aid in preventing denture displacement, a factor that may not be controllable with the passive habituation noted in standard post-insertion care.

#### Biofeedback

The findings by Yemm (1983) suggest that the soreness and mucosal tenderness experienced in some complete denture wearers can be bypassed with simple biofeedback training mechanisms (20). The use of electromyography (EMG) for the same appears to be a simple yet effective mechanism in lowering the resting muscle activity and achieving a more physiologic rest position, aiding in improved patient comfort and denture stability.

### Clinical implications

Although the number of studies included in the present review are limited, their clinical implications are noteworthy. The synthesised evidence indicates that simple oral motor exercises such as reading aloud a newspaper for 15–20 min on a daily basis are a cost-effective, non-invasive, and feasible intervention that can significantly improve the patient's OHRQoL. Similarly, mild biofeedback training via EMG for a mere 10–15 min can achieve effective outputs, a tool which may be considered especially in patients with taut muscles who require muscle relaxation to develop a low resting muscle activity. Unlike the complexity of implant-supported dentures and modified dentures, these interventions can be easily prescribed to any new denture wearer and thus aid in bridging the gap between a technically accurate and a functionally successful denture.

### Strengths and limitations

When it comes to health care interventions, clinical trials provide the best level of evidence, and we followed the recognized guidelines (PRISMA 2020) (16) in this review. To the best of our knowledge, this is the first systematic review to specifically address the benefit of active training interventions over standard post-insertion instruction. The search was extensive and had no restrictions on publication date to include the maximum relevant data and employed dual-reviewer screening to minimize selection bias. However, despite the lack of restrictions, the available literature on this topic was relatively scarce, with only three studies meeting the eligibility criteria and the overall population evaluated was low (*n* = 147). The restriction to English-language articles may have contributed to this by potentially excluding relevant data from other language articles. This limits the generalizability of the findings of the present review. Furthermore, the heterogeneity of the outcome measures prevented a quantitative meta-analysis limitations for publication bias. The possibility of publication bias cannot be excluded. However, formal assessment of publication bias using funnel plots or statistical tests was not considered appropriate because only three studies were included

### Recommendations for further research

The current findings offer valuable preliminary insights into the potential benefits of targeted oral rehabilitation strategies, but the evidence base requires strengthening. Future research should be conducted with a larger sample size to allow for extrapolation, including longer-term interventions that evaluate the lasting effects of neuromuscular re-education, oral motor exercises, or biofeedback compared to standard post-insertion care on masticatory function and patient-reported outcomes. The study designs should be following the protocol of randomized clinical trials, allowing for high-quality evidence to be generated. The training exercises should be of standardized protocols to allow for comparison across studies with longer follow-up periods of 6 months to 1 year to accurately evaluate the prolonged benefits of the interventions beyond the adaptation period.

## Conclusion

The available evidence suggests that neuromuscular re-education, oral motor exercises, and biofeedback may improve aspects of masticatory function and patient-reported outcomes in adults receiving new conventional complete dentures. However, the certainty of evidence is limited by the small number of studies, methodological limitations, and risk of bias. Therefore, these findings should be interpreted cautiously, and further well-designed randomized controlled trials with standardized intervention protocols and longer follow-up are warranted.

## Data Availability

The original contributions presented in the study are included in the article/Supplementary Material, further inquiries can be directed to the corresponding authors.
