# Supplementary material for: The effect of neuromuscular re-education and oral motor exercises on adaptation and masticatory performance in complete denture wearers- a systematic review
Source: Front Dent Med. 2026 Sep 1;7:1949919. doi: 10.3389/fdmed.2026.1949919 (PMC13574555; doi:10.3389/fdmed.2026.1949919)
Supplement: Supplementary file 1 [file Table1.docx]

**PRISMA 2020 flow diagram for new systematic reviews which included searches of databases, registers and other sources**

|  | **Identification of studies via databases and registers** | | |  | **Identification of studies via other methods** | | |
| --- | --- | --- | --- | --- | --- | --- | --- |
| **Identification** | **Records identified from:**  PubMed (n = 165)  Cochrane (n = 183)  Scopus (n = 101)  Web of Science (n = 100)  Google Scholar (n = 968) | **→** | **Records removed *before screening*:**  Duplicate records removed (n = 120)  Records marked as ineligible by automation tools (n = 866)  Records removed for other reasons (n = 0) |  | **Records identified from:**  Citation searching (n = 63)  etc. |  |  |
|  | **↓** |  |  |  | **↓** |  |  |
| **Screening** | **Records screened**  (n = 531) | **→** | **Records excluded****  (n = 527) |  | **↓** |  |  |
|  | **↓** |  |  |  | **↓** |  |  |
|  | **Reports sought for retrieval**  (n = 4) | **→** | **Reports not retrieved**  (n = 0) |  | **Reports sought for retrieval**  (n = 1) | **→** | **Reports not retrieved**  (n = 0) |
|  | **↓** |  |  |  | **↓** |  |  |
|  | **Reports assessed for eligibility**  (n = 4) | **→** | **Reports excluded:**  Ineligible Intervention (n = 1)  Ineligible Population (n = 1) |  | **Reports assessed for eligibility**  (n = 1) | **→** | **Reports excluded:** (n = 0) |
|  | **↓** |  |  |  | **↓** |  |  |
| **Included** | **Studies included in review**  (n = 3)  **Reports of included studies**  (n = 3) | | | | | | |

*Consider, if feasible to do so, reporting the number of records identified from each database or register searched (rather than the total number across all databases/registers).

**If automation tools were used, indicate how many records were excluded by a human and how many were excluded by automation tools.

Source: Page MJ, et al. BMJ 2021;372:n71. doi: 10.1136/bmj.n71.

This work is licensed under CC BY 4.0. To view a copy of this license, visit <https://creativecommons.org/licenses/by/4.0/>
