# Supplementary material for: The effect of neuromuscular re-education and oral motor exercises on adaptation and masticatory performance in complete denture wearers- a systematic review
Source: Front Dent Med. 2026 Sep 1;7:1949919. doi: 10.3389/fdmed.2026.1949919 (PMC13574555; doi:10.3389/fdmed.2026.1949919)
Supplement: Supplementary file 2 [file Table2.docx]

**Characteristics of Included Studies**

| **S.No** | **Study ID (Author, Year)** | **Country** | **Study Design** | **Patient details** | **Total (N)** | **Intervention (N)** | **Comparator (N)** | **Mean Age** | **Sex** | **Intervention Type** | **Intervention Details** | **Intervention Frequency** | **Intervention Session (Duration)** | **Intervention Total Duration** | **Comparator Details** | **Primary outcomes** | **Secondary outcomes** | **Timepoints measured** | **Primary outcomes (results)** | **Secondary outcomes (results)** | **Concluding remarks** |
| --- | --- | --- | --- | --- | --- | --- | --- | --- | --- | --- | --- | --- | --- | --- | --- | --- | --- | --- | --- | --- | --- |
| 1 | Sikandar, et al. (2022) | Pakistan | Randomized Control Trial (RCT) | 60 edentulous patients, 45-54 years, receiving conventional complete denture for the first time. | 60 | 30 | 30 | Group 1 (Intervention): 52.33 ± 6.89  Group 2 (Control): 50 ± 7.87 | Group 1: 52.9% Female  Group 2: 47.1% Female | Oral Motor Exercise (Reading Aloud) | Instructed to read a (Urdu) newspaper in a loud note. | 3 times a day | Increased incrementally: 5 mins/day in week 1 up to 20 mins/day in week 4 | 4 weeks | Standard care. Subjects "did not perform exercises" but were recalled for follow-up. | Masticatory Performance (Sieve method with 3g peanuts, 20 strokes) | N/A | 2 weeks and 4 weeks (post-insertion) | At 4 weeks (Mean % ± SD):  Group 1 (Exercise): 37.13 ± 1.47  Group 2 (Control): 29.60 ± 4.29.  (p < 0.001) | N/A | Masticatory performance can be enhanced after insertion of complete dentures by practicing read aloud exercises. |
| 2 | Yemm R (1983) | Scotland | Clinical Controlled Trial (CCT) | Control group: Edentulous female patients with no history of denture difficulty.  Intervention group: Edentulous female patients who had experienced difficulty with denture soreness.  All female. History of intolerance to lower denture. | 25 | 10 | 15 | Intervention Group: 67  Control Group (Edentulous): 63 | Intervention Group: 100% Female  Control Group (Edentulous): 100% Female | EMG Biofeedback (Visual and Audible) | EMG biofeedback device with visual (20-light column) and audible (click tone) feedback.  Subject was "encouraged to experiment with jaw position" to find lowest muscle activity. | 1 single session | 3 x 5-minute training periods | 15 minutes of training (total) | Two control groups: (1) 10 normal dentate subjects, (2) 5 normal edentulous subjects.  Both control groups underwent the exact same protocol as the intervention group. | Orofacial Muscle Activity (EMG right temporal muscle) | Patient-Reported Outcomes (Subjective reports of comfort) | Baseline (Training time = 0), 5 mins, 10 mins, and 15 mins. (A recall was done 2 weeks later). | Intervention Group (Baseline): 10.8 ± 5.5.  Intervention Group (After 15 min): 3.3 ± 2.3.  (Control groups showed very little change). | Subjective: "two reported feeling comfortable with their lower denture, they thought as a consequence of the relaxation they had practised." | Biofeedback muscle relaxation training may be a useful method of treatment in such cases. |
| 3 | Liu L, et al. (2015) | China | Randomized Controlled Trial (RCT) | Edentulous patients requiring complete dentures for the first time. | 62 | 31 | 31 | Group I (Intervention): 70.0 ± 4.3  Group II (Control): 70.9 ± 4.2 | Group I: 51.6% Female  Group II: 58.9% Female | Oral Motor Exercise (Reading Aloud) | Given a 600-word news report (Chinese) to read aloud.  Advised to gradually increase mouth opening during reading. | 3 times per day (morning, noon, afternoon) | Increased incrementally: 5 mins/day in week 1 up to 20 mins/day in week 4. | 4 weeks | Standard care. Group II "did not perform any reading exercises."  Received standard post-op instructions. | Masticatory Performance (Sieve method with 3g shelled peanuts, 20 strokes) | Patient Satisfaction (VAS 0-10) for 5 items: comfort, esthetics, stability, ability to speak, ability to chew. | 2 weeks and 4 weeks (post-insertion) | At 4 weeks (Mean % ± SD):  Group I (Exercise): 47.60 ± 3.28  Group II (Control): 38.99 ± 3.07.  (p < 0.001) | At 4 weeks: Group I had significantly higher satisfaction for:  Stability (8.87 ± 0.94 vs 7.17 ± 1.00)  Ability to Speak (8.30 ± 0.99 vs 6.97 ± 0.73)  Ability to Chew (7.90 ± 1.00 vs 6.69 ± 1.10).  No diff in Comfort or Esthetics. | Planned reading aloud exercises significantly improved masticatory performance and patient satisfaction with stability, ability to talk, and chew for patients who were fitted with conventional complete dentures for the first time. |
